# Microneedle Mediated Transdermal Delivery of Protein, Peptide and Antibody Based Therapeutics: Current Status and Future Considerations

**DOI:** 10.1007/s11095-020-02844-6

**Published:** 2020-06-02

**Authors:** Melissa Kirkby, Aaron R.J. Hutton, Ryan F. Donnelly

**Affiliations:** School of Pharmacy, Queen’s University, Belfast 97 Lisburn Road, Belfast, BT9 7BL UK

**Keywords:** drug delivery, Microneedle, peptide delivery, protein delivery, transdermal

## Abstract

The success of protein, peptide and antibody based therapies is evident - the biopharmaceuticals market is predicted to reach $388 billion by 2024 [1], and more than half of the current top 20 blockbuster drugs are biopharmaceuticals. However, the intrinsic properties of biopharmaceuticals has restricted the routes available for successful drug delivery. While providing 100% bioavailability, the intravenous route is often associated with pain and needle phobia from a patient perspective, which may translate as a reluctance to receive necessary treatment. Several non-invasive strategies have since emerged to overcome these limitations. One such strategy involves the use of microneedles (MNs), which are able to painlessly penetrate the *stratum corneum* barrier to dramatically increase transdermal drug delivery of numerous drugs. This review reports the wealth of studies that aim to enhance transdermal delivery of biopharmaceutics using MNs. The true potential of MNs as a drug delivery device for biopharmaceuticals will not only rely on acceptance from prescribers, patients and the regulatory authorities, but the ability to upscale MN manufacture in a cost-effective manner and the long term safety of MN application. Thus, the current barriers to clinical translation of MNs, and how these barriers may be overcome are also discussed.

## Introduction

The increasing development and use of protein based therapies over the last few decades can be attributed to the improvement of protein expression and synthesis on the scale required for widespread manufacturing (1,2). Protein and peptide based drugs are now widely available and are considered first line treatments for a number of chronic health conditions, such as type I diabetes, rheumatoid arthritis, specific cancers and haemophilia (3). Proteins, peptides and antibody based therapeutics have the potential to treat diseases that were once thought incurable (4) and thus continue to be studied despite ongoing difficulties associated with their delivery.

Protein and peptide based drugs are primarily administered via the parenteral route, as this provides rapid drug delivery, and in the case of the intravenous route, 100% bioavailability. Such high bioavailability is required particularly for proteins and peptides because of the potential for rapid degradation and clearance once in the bloodstream (5,6). Although antibody therapies may be modified to extend their circulatory time in the body, very large doses are still required to provide a therapeutic effect, making the parenteral route the most practical route of delivery. Further properties typically associated with protein and peptide based drugs, such as a high molecular weight and poor tissue membrane permeability, limit the administration route and bioavailbility available via other routes (7,8). For example, delivery via the oral route is hindered by the presence of protease enzymes in the gastrointestinal tract, which readily denature proteins.

However, long-term administration of protein and peptide based drugs via the parenteral route is not without its disadvantages and complications, despite it being the traditional method. From a patient perspective, repeated intravenous drug delivery may be associated with needle phobia, pain, and more complex issues, for example phlebitis and tissue necrosis (1,9). The need for repeated administration due to rapid clearance from the blood increases the risk of toxic adverse effects (10,11). Proteins and peptides present in infusions may also trigger an immune response if the body recognises them as antigens (12,13).

To improve the bioavailability and stability of protein, peptide and antibody based drugs, alternative routes of administration have been sought. Ideally, these routes should allow patients to self-administer the drug and therefore should be minimally invasive and ideally, painless. Additionally, the route should allow rapid onset of drug action with potential for sustained drug delivery, to reduce the need for repeated administration. Alternative administration routes investigated include the pulmonary (14–16), ocular (17–19), nasal (14,20–22), rectal (22–24) and transdermal (25–27) routes. The justification for each of these administration routes have been summarised in detail elsewhere (28) and each possess advantages and disadvantages, which are summarised in Table I.

**Table I:** Advantages and disadvantages of administration routes for protein, peptide and antibody based therapeutics. Created from information provided in (28)

Route of administration	Advantages	Disadvantages
Parenteral	Intravenous route offers 100% bioavailability Rapid delivery of drug into systemic circulation Viable alternative if oral route is not feasible	Intravenous route is painful, invasive and poorly tolerated by patients Potential for toxic effects due to repeated administration
Oral	Painless Convenient	Potential for poor permeability across the intestinal epithelial membrane First pass metabolism Proteases present in the gastrointestinal tract may degrade drug
Pulmonary	Painless Large surface area available for protein absorption Avoids first pass metabolism Low enzyme activity in the lungs	Potential for poor permeability across epithelial lining fluid, epithelial cell layer and the endothelial membrane of capillary cells Proteins and peptides may be subjected to phagocytosis by the macrophages in the lungs
Ocular	Avoids first pass metabolism	Potential for poor permeability, particularly of hydrophilic macromolecules, across eye membrane High enzyme activity, i.e. protease and aminopeptidase
Nasal	Painless Large surface area available for protein absorption Avoids first pass metabolism Thin porous endothelial basement membrane of the nasal epithelium facilitates drug absorption	Potential for poor permeability, particularly of large hydrophilic macromolecules, across nasal epithelium Rapid mucociliary clearance that reduces the available time for drug absorption Only small amounts of drug can be administered via the nasal route
Rectal	Offers partial bypass of first pass metabolism	Potential for poor permeability across rectal epithelium Patient may consider this route distasteful
Transdermal	Painless Convenient Large surface area available for protein absorption Avoids first pass metabolism Potential for adaptability to deliver both small and macromolecular therapeutics, e.g. by using microneedles	Potential for poor permeability, particularly of large hydrophilic molecules, across the *stratum corneum* Potential for localised skin irritation

### Microneedles (MNs)

**Fig. 1:**
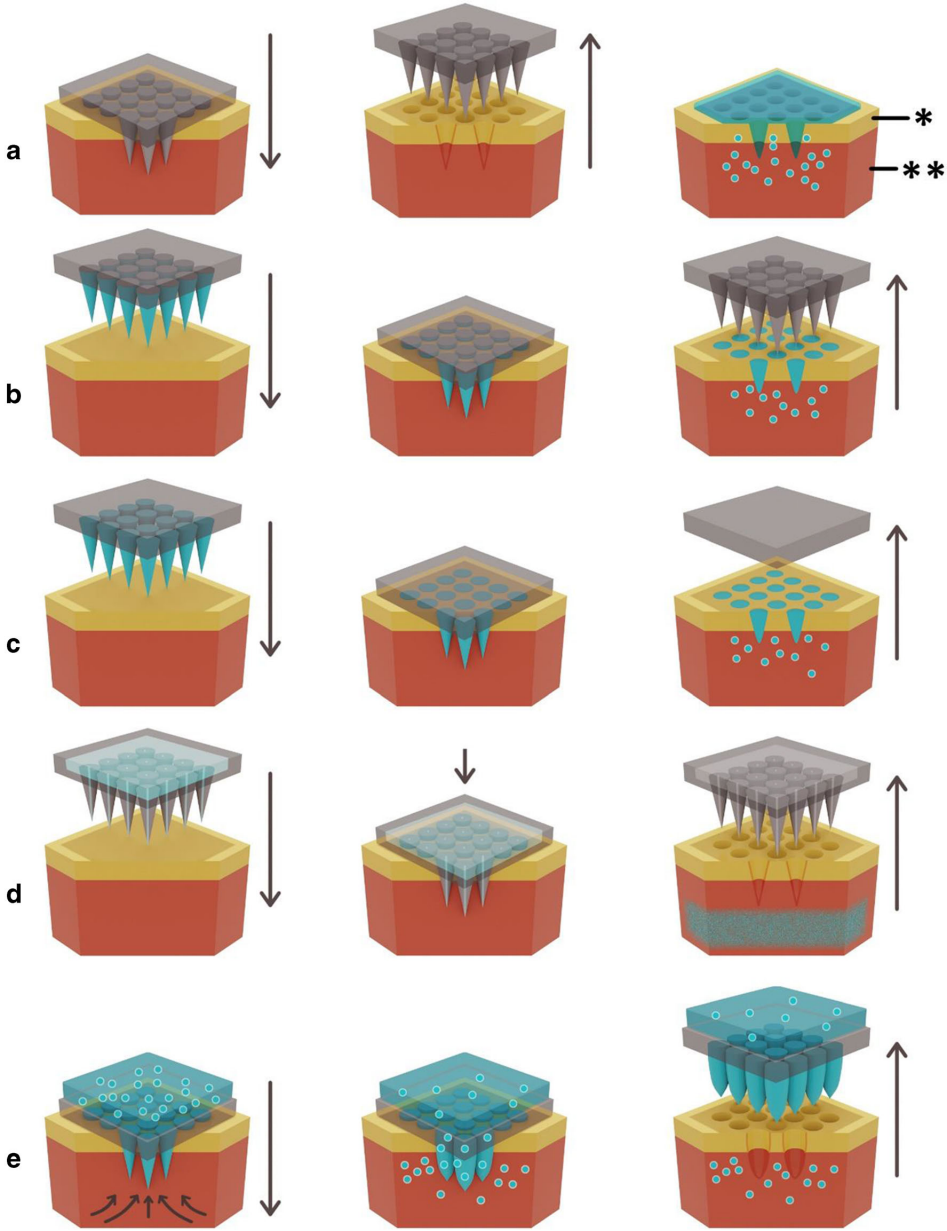
Schematic representation of methods of MN application to the skin to achieve enhanced transdermal drug delivery, * stratum corneum, ** epidermis. (A) Solid MN that are applied and removed to create transient micropores, followed by application of the formulation. (B) Solid MN are coated with drug for instant delivery and to remove the two step process associated with solid MNs. (C) Drug is mixed with soluble polymeric/carbohydrate MNs that dissolve in skin interstitial fluid over time. (D) Hollow MNs puncture the skin, after which liquid drug can be actively infused through the needle bores. (E) Hydrogel-forming MNs imbibe skin interstitial fluid upon application to the skin. This induces drug diffusion through the swollen microprojections. Drug is often stored above the microprojections in a lyophilised wafer prior to interstitial fluid uptake

Microneedle (MN) arrays consist of multiple micro-projections assembled on one side of a supporting base, ranging in height from 25 to 900 μm. MN arrays effectively bypass the *stratum corneum* barrier by creating temporary microscopic aqueous channels within the epidermis, through which drug molecules can diffuse into the dense microcirculation, present in the dermis. MNs were first conceptualised by Gerstel and Place in 1971 (29), but were not practically realised until 1998, when manufacturing capabilities and microfabrication techniques became more advanced. Today, MN technology has developed further and they are traditionally placed in five different categories: solid, coated, hollow, dissolving and hydrogel-forming (Fig. 1).

Each type of MN has its own distinct advantages and disadvantages and therefore it is important to determine the type of MN required for maximised transdermal delivery of a specific drug. Solid MNs may be combined with any conventional drug formulation for passive diffusion (i.e. transdermal patch, solution, cream or gel), however, the two-step application process is more impractical than other methods and may discourage patient use.

The use of coated MNs removes the two-step application process, however, the finite surface area of the needle array limits the amount of drug that can be applied. Thus, coated MNs are typically limited to use with potent drugs.

Dissolving MNs use biocompatible polymers mixed with the drug to form the needle tips. As the needle tips dissolve once applied to the skin, there is no risk of accidental re-piercing of the skin and no need for sharps disposal, a potential problem associated with solid, coated and hollow MNs. Additionally, dissolving MNs provide potential for controlled drug release - the release kinetics of the drug are dependent upon the constituent polymers’ dissolution rate (30). By adjusting the type of polymer and the polymer composition within the formulation, drug release may be controlled. Typical polymers used for the production of dissolving MNs include poly(vinyl alcohol) (PVA), poly(vinylpyrrolidone) (PVP), dextran, carboxymethyl cellulose (CMC), chondroitin sulfate and various sugars (31), all of which are low cost and therein lies the potential for cheap and straightforward mass production. The main limitation associated with dissolving MNs is the deposition of polymer, alongside the drug, into the skin. Although polymers discussed above are biocompatible, currently no long-term studies explore the effects of repeated polymer deposition into the skin. Further, long term research will be required to provide safety assurances to both prescribers and patients (32).

As an alternative, biodegradable polymers, such as poly(lactic acid), chitosan, poly(glycolic acid), or poly(lactide-co-glycolide) (PLGA), have been explored, which degrade, rather than dissolve, to release the drug. Carbohydrates have also been used as a dissolving MN material. They are cheap, safe, and can sufficiently pierce the skin (33–35). However, several problems associated with their processing and storage prevent their use clinically (36), primarily thermal treatment required during the manufacturing process which limits the number of drugs available for loading into the MN arrays.

Hollow MNs allow a greater volume of drug to be delivered into the skin, either by passive diffusion, or by infusion using pressure or electricity to drive the direction of drug flow into the skin (37). However, this may require bulky associated equipment (such as an electronic pump with associated electronics and microprocessor), reducing the convenience associated with MNs to a certain degree. The primary disadvantage associated with hollow MNs is the potential for drug flow resistance to occur – either by clogging of needle openings with skin tissue during insertion (38), or by compression of the MNs by dense dermal tissue (39). Limitations may be overcome somewhat by use of an alternative MN design (40), or by partially removing MNs immediately following insertion to reduce tissue clogging at the needle tips (41). Stability issues may arise when using hollow MNs, as the drug must be in liquid form for delivery. For biomolecules specifically, this would likely require a cold chain to be maintained from bench to bedside to ensure biomolecule stability. This removes the advantage associated with other MN types, for example, hydrogel-forming MNs, whereby the drug may sit in a compressed tabled or lyophilised wafer above the array until the array is applied.

Hydrogel-forming MNs are the most recent type of MN to be formulated (42,43). MNs used in this system do not contain drug, and instead integrate cross-linked polymeric MN projections with an attached drug reservoir. Figure 1E demonstrates that following interstitial fluid uptake, the drug may diffuse from the reservoir, through the swollen MNs, into the skin, to be up-taken by the dermal microcirculation. The most common types of polymeric materials used in the aqueous hydrogel blend include poly(methyl vinyl ether-co-maleic acid) crosslinked by esterification using poly(ethyleneglycol), chitosan, PLGA and PVA (32,42,44,45). Similarly to dissolving MNs, the delivery of drug may be controlled by the polymer blend, which affects the cross-linking ratio. Cross-linking hinders the mobility of the polymer chains and therefore reduces the swelling abilities of the hydrogel. There is the potential for interactions to occur between the hydrogel matrix and drug; therefore, drugs must be tested on an individual basis to determine their compatibility with the polymers used in the hydrogel-forming MN system. More recently, hydrogel-forming MNs have been made from light responsive polymer materials to control drug release (46). A further advantage of hydrogel-forming MN arrays is the fact that they are removed intact from the skin. Therefore, there is no concern with polymer left in the skin, as is the case with dissolving MNs. As the MNs are swollen, they cannot be re-inserted into the skin, removing the risk of accidental re-insertion and the need for sharps disposal.

### Materials for MN Fabrication

**Fig. 2:**
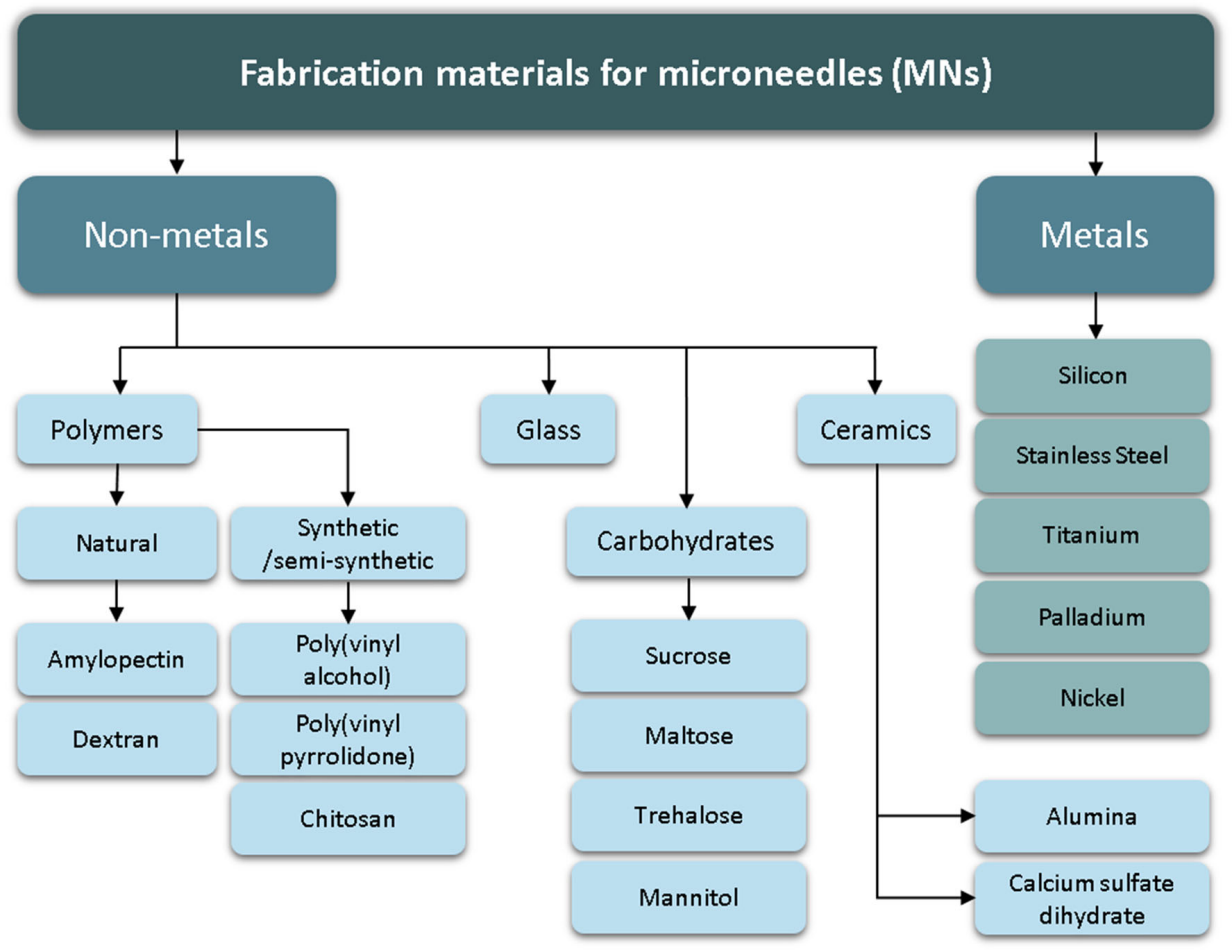
Materials used for the preparation of MNs

As briefly discussed above, the type of material used to make MNs in dissolving and hydrogel-forming systems will influence the drugs ability to diffuse into the skin. There has been numerous studies that explore the material types used to create MNs and their biocompatibilities. MNs were initially manufactured from silicon (47), but have since been made from materials such as stainless steel (48), silk (49) and various polymers (50,51) (Fig. 2).

Silicon was the first material used for MN fabrication, prior to the development of more complex fabrication techniques (38,52). It is a versatile material, able to be fabricated into a range of MN shapes, with suitable strength to pierce the skin (53). The limitations associated with silicon MNs are the high cost associated with its use, including long fabrication times and multi-step processing. This increases the cost associated with silicon MN use, although MNs formed from this material can be made in batches to reduce costs (54). Furthermore, some concerns exist regarding the biocompatibility of silicon, as the needles may become brittle once fabricated, increasing the risk of material fracture when piercing the skin. The failure of the first MN device, Micronject® (Fig. 4G), may be attributed to some extent to its silicon needles, which are not biodegradable and may cause biofouling (56,57). Chen et al. (2008) produced silicon MNs with biodegradable tips in an attempt to negate this issue. Silica glass has also been investigated as a MN material (58). Although the material is inert and its transparency allows visualisation of fluid flow (41,59), the material is brittle and has similar fracture toughness to silica (54).

Various types of metals, such as stainless steel, titanium, palladium, palladium-cobalt alloys and nickel have been used to fabricate MNs (60). Metals are an attractive material for MN fabrication as their use is established in healthcare, for example, stainless steel hypodermic needles and titanium implants. Metals used for MN fabrication exhibit good biocompatibility and high fracture forces, reducing the risk of needles breaking off in skin tissue (61).

Ceramic MNs are typically fabricated by casting ceramic slurries into micromoulds (55), a low cost process with the potential for up-scaling. Types of ceramic used include alumina (Al_2_O_3_), calcium sulfate dihydrate (CaSO_4_·2H_2_O) and calcium phosphate dihydrate (CaHPO_4_·2H_2_O) (62,63). Alumina in particular is resistant to corrosion and adverse environmental conditions (64). Ceramics typically have good compression resistance, but can be brittle when exposed to tensile stress (62), and ultimately, have poorer strength than alternative materials such as metals.

To summarise, MNs are a minimally invasive drug delivery device, which combines the benefits of a transdermal patch with the drug delivery capabilities of a hypodermic needle. Their application is painless with minimalised skin trauma and bleeding compared to that of a hypodermic needle, a highly attractive attribute for patients (65,66). In addition to a reduction in needle phobia and reduced risk of infection, MNs can be self-administered, removing the need for healthcare staff support (67,68). Furthermore, dissolving and hydrogel-forming MNs eliminate the need for sharps waste disposal. It can be expected that these patient-friendly benefits of MNs may be translated into increased compliance. However, the benefits of MNs are not limited to the patient. There has been a large amount of research pertaining to the removal of the “cold chain” through vaccine-MN manufacturing, which would result in huge cost savings if accomplished (69,70). Numerous parameters can be changed to provide the most efficacious and controlled delivery of a specific drug, bypassing first pass metabolism and allowing the delivery of both small molecules and macromolecules. MNs hold the potential to transform transdermal drug delivery. There have been numerous studies demonstrating the drug delivery capabilities of solid (50,52,71–74), coated (75–78), dissolving (34,79–81), hollow (59,82,83), and hydrogel-forming (32,84–88) MNs. This review will focus on MN-mediated delivery of protein, peptide and antibody based therapies, and the hurdles that must be overcome for MNs to be accepted for clinical use.

## MN Mediated Transdermal Delivery of Protein, Peptide and Antibody Based Therapeutics

### Solid Microneedles

Diabetes affects 422 million people worldwide (89). The first line therapy for type 1 diabetics is daily subcutaneous insulin injections, and commonly becomes a later necessity for the treatment of type 2 diabetes. Therefore, alternative insulin delivery methods have become a popular route of scientific exploration. McAllister et al. (2003) fabricated solid silicon MNs to facilitate the delivery of insulin and bovine serum albumin (BSA) across human skin in vitro (50). Permeation of the two compounds was successful, and permeability of both was increased compared to when MNs were left in the skin, demonstrating that the compounds were able to diffuse through the aqueous channels created by the silicon MNs. The concentration of insulin delivered across the skin from a 1 cm^2^ patch containing 100 units/mL was deemed sufficient to meet the basal needs of many diabetics.

A secondary study exploring the ability of solid MNs to deliver insulin transdermally was completed by Martanto et al. (2004). Similarly to McAllister et al. (2003), MNs increased skin permeability to insulin, to an extent equal to a 0.05–0.5 units of insulin injected subcutaneously. Blood glucose levels in diabetic rats were lowered by as much as 80% (71).

Zhou et al. (2010) investigated the effects of differing metal MN lengths (250 μm, 500 μm and 1000 μm) on the transdermal delivery of insulin. For all three needle lengths, blood glucose levels rapidly decreased in 1 h and continued to decrease until 3 h. Glucose levels slowly increased thereafter, this was associated with the closure of the temporary micropores created by the MNs, confirmed by transepidermal water loss (TEWL). Furthermore, the rate of elevation in blood glucose levels was inversely proportional to the length of the needle (90).

More recently, Li et al. (2017) created solid MNs from poly(lactic acid) (PLA) to combine the advantages of MNs with the added benefit of a biodegradable system, an attractive prospect from a commercial point of view. The study systematically investigated the effects of MN dimensions, drug (insulin) concentration, viscosity of drug formulation and the administration time of drug on its transdermal delivery. Increasing insulin concentration increased the permeation amount, but not rate, of drug in vitro. Increasing formulation viscosity decreased permeation rate. In vivo studies were then conducted on diabetic mice, using solid PLA MNs with a height of 600 μm and a density of 100 MNs per cm^2^. The minimal blood glucose levels were found to be 29% at 5 h, compared to 19% at 1.5 h from a subcutaneous insulin injection. The authors concluded that the use of MNs may be beneficial when a delayed reduction in blood glucose is required (91).

Solid MN studies are not limited to the delivery of insulin. For example, Li et al. (2010) investigated the effects of solid MN (metal DermaRoller™ and maltose) pre-treatment on the transdermal delivery of human immunoglobulin G (IgG) in vivo (5 mg/mL applied concentration). Flux was recorded as 45.96 ng/cm^2^/h and 353.17 ng/cm^2^/h in vitro for maltose and metal MNs respectively. C_max_ was recorded as 7.27 ng/mL and 9.33 ng/mL at 24 h for maltose and metal MNs respectively. The ability of the DermaRoller™ to create wider MN channels was attributed to the increase in both flux and C_max_ (92).

Cui et al. (2011) evaluated the extent to which pre-treatment with MNs (DermaRoller™, 250 μm, 500 μm and 1000 μm) could enhance skin permeation of ovalbumin-conjugated nanoparticles in vitro and in vivo. For in vitro studies, MN pre-treatment increased ovalbumin permeation significantly more than the control. Furthermore, 28.3 ± 6.5% of ovalbumin was delivered transdermally from ovalbumin in solution compared to 13.6 ± 2.4% ovalbumin delivery from ovalbumin nanoparticles. This was attributed to the greater size of the ovalbumin nanoparticles hindering diffusion. When applied as a 70 μg/mouse dose, transcutaneous immunisation from ovalbumin nanoparticles following MN pre-treatment was greater than the same dose given subcutaneously (93).

Han and Das (2013) combined sonophoresis and MNs to enhance the delivery of BSA across porcine ear skin. Permeability of BSA was found to be 0.43 and 0.40 μm/s from MNs and sonophoresis alone, however, when the two physical methods of permeation enhancement were combined (1.5 mm MNs, 15-W ultrasound), permeability increased to 1 μm/s. This was reported as approximately 10 times higher than that achievable by passive diffusion of BSA (94).

Zhang et al. (2014) determined transdermal permeation of four model peptides following a 150 μm solid silicon MN pre-treatment across porcine ear skin. Similarly to Cui et al. (2011), molecular weights of the peptides influenced their ability to permeate transdermally. MN pre-treatment significantly enhanced permeation of all peptides, although increasing the molecular weight of the peptides decreased the amount delivered transdermally (95).

### Coated MNs

Many studies that use coated MNs focus on the field of vaccination (96). Minimal amounts of vaccine delivered into the skin can still generate the required immune response, due to the high levels of Langerhans and dendritic cells within the skin (97). As minimal vaccine is sufficient, the reported disadvantage of limited drug loading on coated MNs does not apply, hence the popularity for using coated MNs for vaccine delivery. The use of MNs for vaccine delivery has been reviewed elsewhere in depth (31). This review will focus on delivery of proteins and peptides for therapeutic, rather than immunological benefits.

Saurer et al. (2010) successfully coated stainless steel MNs with DNA and protein-containing polyelectrolyte films in a layer-by-layer approach. The authors cited five key advantages of using these types of films – there is precise control over film thickness and therefore drug concentration; organic solvents are not required in the fabrication process, improving the safety of the MNs; fabrication of films provides control over the release of defined amounts of multiple different agents; auxiliary agents may be incorporated into the films (e.g. cationic polymers); and the fabrication process is able to coat objects having irregular shapes such as medical devices or implantable materials. In this study, the release of both protein and DNA from the coated MNs was characterised by fluorescence and optical microscopy following 2 h insertion into porcine cadaver skin. Post insertion fluorescence images demonstrate the capability of the coated layer to be released almost completely from the solid MNs and to be delivered into the epidermal and dermal layers of skin (98).

Acknowledging that MN mediated drug delivery focused mainly on hydrophilic molecules, Zhao et al. (2017) developed a novel formulation for the coating of MNs for delivery of hydrophobic auto antigen peptides, which are being investigated for antigen specific immunotherapy of type 1 diabetes. The formulation was comprised of three co-solvents (water, 2-methyl-2-butanol and acetic acid) and PVA 2000, which could dissolve both hydrophilic and hydrophobic peptide auto-antigens at relatively high, and clinically relevant, concentrations. The formulation coating and procedure did not adversely affect the biological activity of the peptides. Both in vitro (human skin) and in vivo (mouse skin) studies were completed to demonstrate the ability of hydrophobic peptides to be delivered via coated MNs. Delivery was maximised when electropolishing the underlying metal MN array, reducing the thickness of peptide coating and utilising peptides with greater aqueous solubility (99).

Caudill et al. (2018) utilised PEG MNs for the delivery of BSA in vitro and in vivo. MNs were inserted into a solution-filled coating mask device, then withdrawn and allowed to dry before piercing the skin. In vitro permeation of FITC-BSA loaded MNs (1000 μm, 64 needles/cm^2^) across full thickness porcine skin following 5 min MN insertion was found to be 45% at 24 h. FITC-BSA appeared to be concentrated in the epidermis, upper layers of the dermis, and around sites of microneedle penetration, with little fluorescent signal observed in the lower dermis. The effectiveness of the MNs was attributed to the needle density and needle length (100). The authors followed up the in vitro data with an in vivo study. MNs (700 μm in height) were coated with a 7% BSA solution and applied to the back of BALB/c mice for 2 min. Compared to a control subcutaneous dose, MN treated mice showed a more sustained retention of BSA at the site of administration. Furthermore, BSA was retained within the skin for a greater time period than the subcutaneous dose. MN treated mice had 79% and 19% fluorescence signal remaining at 6 h and 72 h, respectively. This is compared to the subcutaneous dose, which resulted in 14% and 4% fluorescence signal remaining at 6 h and 72 h, respectively. This depot effect was attributed to the presence of high molecular weight methylcellulose (MW 17,000 Da) within the MN formulation, which retained the coated BSA near the administration site for greater periods of time.

Li et al. (2018) coated the surface of individual metal MNs with various compounds (immiscible molecules, proteins, and nanoparticles) to allow delivery of a variety of therapies within the same MN patch. The compounds chosen represented drugs of different sizes and both particles and free drugs, in order to represent almost any type of therapy which may be utilised within MN systems. MNs were applied to full thickness porcine skin for 5 s and removed after 2 min. The protein used in the in vitro experiment was FITC-BSA, and was delivered alongside free fluorescein sodium dye and fluorescently labelled nanoparticles. Results showed that all three compounds were successfully delivered, but at differing rates. FITC-BSA delivery sat between the three compounds, with fluorescein sodium dye diffusing the fastest and fluorescently labelled nanoparticles diffusing the slowest. FITC-BSA fluorescent intensity declined to ~40% after 4 h and trace remains were left at the end of the 2 day experiment (101).

### Dissolving MNs

Similarly to coated MNs, dissolving MNs have been investigated extensively for vaccine delivery (102–104), and their advantages, discussed earlier in this review, continue to support their exploration for delivery of protein and peptide drugs.

Mönkäre et al. (2015) developed monoclonal IgG loaded hyaluronan-based dissolving MNs for intradermal delivery in vitro. Following 10 min application to human skin (280 μm length), the majority of the original tip length (65%) was dissolved and IgG and hyaluronan were co-deposited until a depth of 150–200 μm in the skin. The authors noted that the low molecular weight of the hyaluronan likely improved the dissolution rate compared to other studies using hyaluronan for the basis of their dissolving MNs (105).

Chen et al. (2016) formulated interferon-α-2b containing dissolving MNs (680 μm length) for transdermal drug delivery. In vitro drug release efficiency was 49.2%. In vivo studies reported a C_max_ and T_max_ of 11.58 ng/mL at 40 min. The dissolving MNs showed sufficient stability for 2 months. The authors reported that the bioequivalence was similar between dissolving MNs and an intramuscular (IM) injection control, suggesting that IM injections of interferon-α-2b could be replaced with MNs for self-administration and increased patient compliance (106).

A dissolving MN system, comprised of PVA and trehalose to encapsulate active pharmaceutical peptides within the MN matrix was created by Dillon et al. (2017). Polymyxin B loaded MNs were applied to porcine ear skin for 30 s. The rate of drug delivery was found to be greater than the control (drug loaded disc without MNs) for the first 4 h post MN application, after which rate of permeation was equal to the control, but the percentage of drug delivered transdermally was significantly greater. At the end of the 22 h Franz cell experiment, 66.9 ± 11.59% of polymyxin B was delivered transdermally, compared to 54.14 ± 3.01% for the control (107).

To remove the two step application of solid MNs, Liu et al. (2018) fabricated insulin-loaded dissolving MNs for glucose regulation in diabetic rats. A two-step centrifuging and moulding process was used to form a dissolving composite containing insulin-loaded CaCO_3_ microparticles and PVP. Each patch contained 10 × 10 array of needles, 250 μm needle length. When compared to pure PVP MNs, mechanical strength was increased and solubility was slower, providing controlled release properties. Similarly to the study completed by Li et al. (2017) discussed above, delivery of insulin from the MNs was slower than that of a control subcutaneous injection. The 5 IU subcutaneous injection lowered blood glucose levels to 29.5 ± 5.2 mg/dL 2 h post injection, compared to 39.7 ± 7.5 mg/dL at 5 h post MN insertion containing the same units of insulin (108).

It is clear that numerous parameters can be changed to optimise the stability and activity of drugs encapsulated within a dissolving MN system. Lahiji, Jang, Huh, et al. (2018) and Lahiji, Jang, Ma, et al. (2018) evaluated the effects of polymer type, concentration, drying conditions and storage temperature on the activity of lysozyme (model protein) loaded in dissolving MNs. The activity of lysozyme was preserved up to 99.8 ± 3.8% for 12 weeks when fabricated at 4°C, allowed to dry naturally and when fabricated within the presence of stabilising agents such as trehalose (109,110).

Vora et al. (2020) acknowledged that there is a limited range of water soluble, biodegradable polymers that can be used to manufacture dissolving MNs. They therefore used a carbohydrate biopolymer (pullulan) for the first time to facilitate delivery of FITC-BSA across dermatomed neonatal porcine skin. After assuring stability of FITC-BSA remained intact in the formulation, in vitro studies were used to assess transdermal delivery of FITC-BSA from the novel dissolving MNs (600 μm needle length). FITC-BSA was detectable as soon as 15 min post-MN insertion, and at 28 h, 1105 ± 123 μg/cm^2^ was delivered from the dissolving MNs. Therefore, the authors demonstrated for the first time the potential of the carbohydrate biopolymer pullulan for fabrication of dissolving MNs for the successful delivery of high molecular weight compounds such as FITC-BSA (111).

### Hollow MNs

Most studies regarding hollow MN arrays have focused on fabrication aspects, including design and characterisation studies. As a result, less attention has been given to their actual efficiency in delivering drug molecules across the skin (112). Again, focus has been given to intradermal delivery of vaccines, particularly those loaded in nanoparticles, which could not be delivered by other means i.e. coated MNs (113,114). Comparison with dissolving MNs has also occurred (115).

Delivery of high molecular weight compounds into the skin was questioned by Chen et al. (2010). The authors believed the answer to this question might lie in the combination of sonophoresis, a technique that uses low frequency ultrasound to induce acoustic cavitations in the lipid layers of the SC, and MNs. The transdermal delivery of calcein and BSA was measured passively, with either sonophoresis or hollow MNs (300 μm length) alone, or when the two methods were combined (SEMA). For both compounds, transdermal delivery was in the order of; SEMA > sonophoresis alone > hollow MNs alone > passive diffusion (116). Although the study effectively demonstrated that the two physical methods of permeation enhancement could increase transdermal delivery of macromolecules, the addition of sonophoresis to MNs removes some key advantages of MNs, namely the ability for self-administration and the convenience associated with the small array. The addition of sonophoresis also returns the device to a two-step process, similarly to the use of solid MNs.

A “pocketed” MN device design was created by Torrisi et al. (2013) for the intradermal delivery of botulinum toxin A to reduce pain, improve therapeutic targeting and to streamline the administration procedure. Pockets were cut into stainless steel MN shafts for liquid drug reservoir loading. Microneedle-mediated intradermal delivery of β-galactosidase and formaldehyde-inactivated botulinum toxoid revealed effective deposition and subsequent diffusion within the dermis (117).

In vitro intradermal delivery of synthetic mRNA using hollow MNs was demonstrated by Golombek et al. (2018). High levels of humanised Guassia luciferase (hGLuc) protein were detectable following hollow MN penetration. Levels after 24 h and 48 h were significantly higher than the control “naked mRNA” (118).

### Hydrogel-Forming MNs

Hydrogel-forming MNs are a relatively newer type of MN compared to those discussed above (42). Thus, many studies have focused on changing parameters that may affect their swelling capabilities, and therefore, their ability to deliver drugs transdermally. Factors affecting transdermal drug delivery from hydrogel-forming MNs include polymer content (119), molecular weight of the cross-linking agent (120), concentrations of the cross-linking agent (121) and presence of foaming agent (122). Electrical modulation via iontophoresis (ITP) combined with hydrogel-forming MNs has also been shown to enhance transdermal delivery (40). Using this approach, Donnelly et al (2012) deemed it possible to facilitate on demand requirements, such as the delivery of insulin after a meal or rapid vaccine delivery. However, enhancing the delivery of proteins and antibody-based therapeutics is limited due to the approximated 13 kDa molecular limit associated with ITP delivery (121,122).

To improve adhesion to the skin, Seong et al. (2017) formulated double layered MN arrays with swellable needles inside a non-swelling patch, which are able to interlock upon skin insertion. This interlocking behaviour was attributed to the sustained release of insulin in vivo. Over 12 h, 60% of the applied insulin was delivered transdermally, 70% of which had a stable confirmation. The authors suggest this novel MN design could be used in the future where sustained release kinetics are required (123).

Courtenay et al. (2018) compared dissolving and hydrogel-forming MNs (500 μm needle length) for the transdermal delivery of bevacizumab in vivo. The dissolving MNs delivered a higher C_max_ at a faster rate (488.7 ng/mL at 6 h) compared to the hydrogel-forming MNs (81.2 ng/mL and 358.2 ng/mL at 48 h for the hydrogel-forming MNs containing 5 mg and 10 mg of bevacizumab respectively). The differences in the pharmacokinetic profile was attributed to the molecular weight of bevacizumab (149,000 Da). It was suggested that diffusion of the large macromolecule through the tortuous hydrogel network was likely to have been the cause of the delayed C_max_ compared to dissolving MNs (88). PVA is a hydrophilic polymer, and thus bevacizumab incorporation into PVA dissolving MNs would allow immediate dissolution and drug release upon MN insertion. As it appeared that the drug was released as a bolus from dissolving MNs, but a sustained release profile was observed from hydrogel-forming MNs, the MN type could be tailored to the desired pharmacokinetic profile for the delivery of high molecular weight macromolecules.

## Safety and Clinical Translation of MN Based Products for Protein, Peptide and Antibody Based Therapeutics

**Fig. 3:**
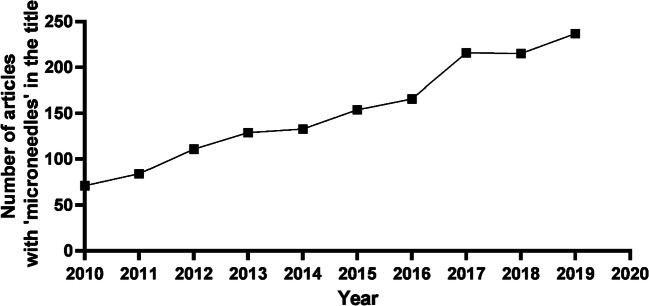
Number of journal articles published containing ‘microneedle’ in the title each year since 2010 (data acquired from PubMed)

The field of MNs has grown immensely since they were first conceptualised by Gerstel and Place in 1971 (Fig. 3). Extensive studies have explored MN fabrication and drug loading techniques to optimise the system. In more recent years, delivery of high molecular weight, high dose and low potency protein and peptide based therapies has become more commonplace, allowing MNs to be considered for delivery of drugs which was previously thought unlikely or even impossible.

**Fig. 4:**
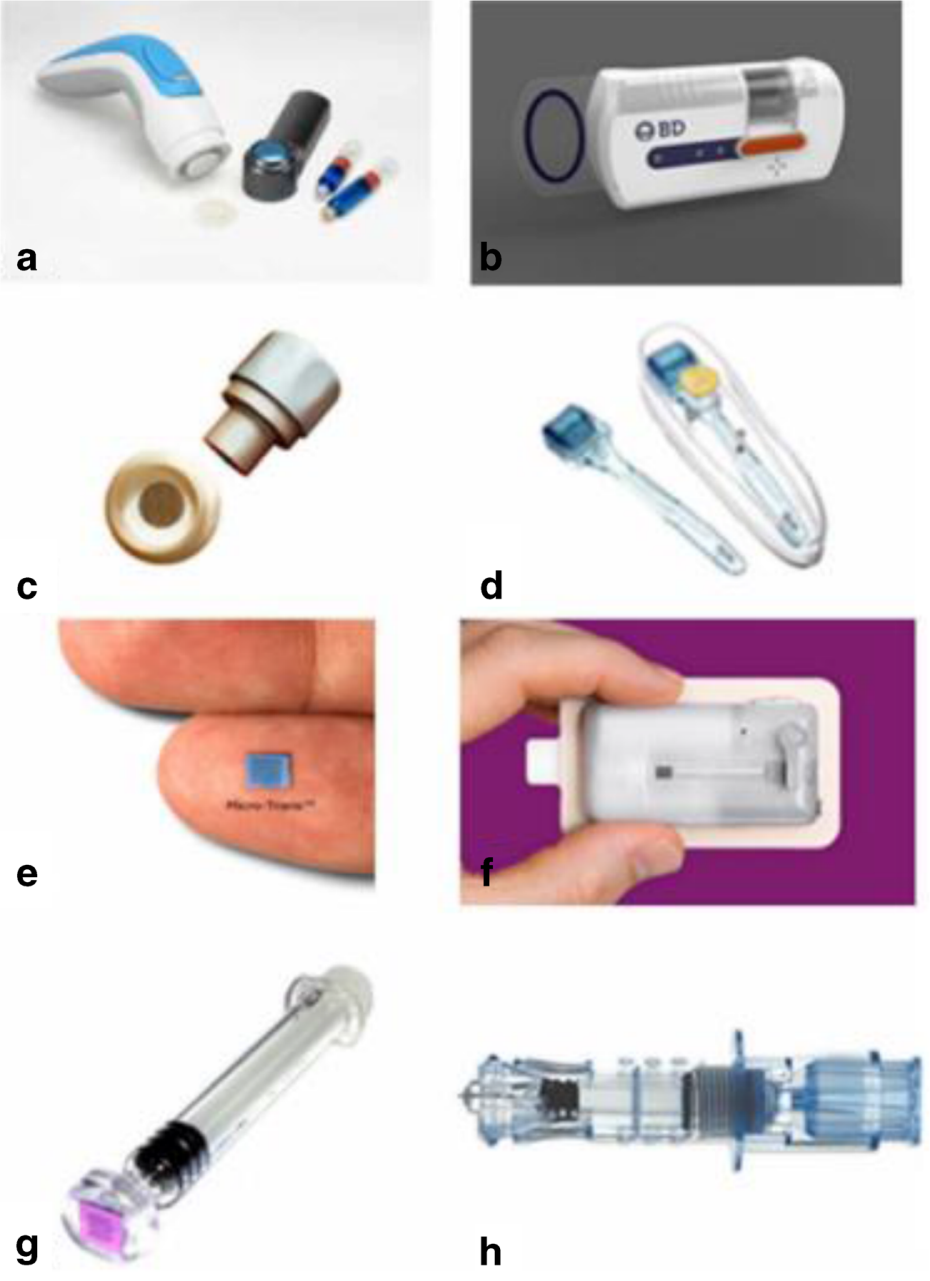
*Current microneedle devices. A Microstructured Transdermal System (MTS). B Microinfusor. C Macroflux®. D Microneedle Therapy System (MTS Roller™). E Microtrans™. F h-patch™. G MicronJet. H Intanza®. Reproduced with permission from* (55)

The transdermal drug delivery market is predicted to grow by $1.79 billion between 2019 and 2023 (124), and the biopharmaceuticals market is predicted to reach $388 billion by 2024 (125). Biopharmaceuticals can technically modulate any physiological pathway that has been fully understood, thus, there is a huge growth potential. More than half of the current top 20 blockbuster drugs are biopharmaceuticals, illustrating the growth and interest in both the biopharmaceuticals and transdermal market. The next step for clinical success is clinical trials. A ClinicalTrial.gov search reports 106 studies for the keyword *microneedle* (March 2020), 67 of which have been completed worldwide. Studies that focus on intradermal vaccination, diabetes and anaesthesia are the most common. Of the completed studies, only four have reached Phase IV trials, one of which involves the intradermal delivery of the influenza vaccine. However, the use of protein, peptide and antibody based therapies transdermally faces numerous challenges that must be addressed before they can achieve their full potential.

### Patient Safety

MNs now harbour the ability to deliver drugs that require high doses and are of low potency (126), as opposed to the traditional delivery of low dose, high potency therapies (127,128). Piercing the skin using MNs results in significantly lower microbial penetration than that produced by using a conventional hypodermic needle (72,129), and hydrogel-forming MNs have even demonstrated antimicrobial properties (130). Therefore, the likelihood of MNs inducing a skin or soft tissue infection is minimal. Furthermore, it is statistically unlikely that MNs will ever pierce the exact same points on the skin surface due to the small size of the device, increasing the likelihood of MNs having a favourable safety profile (131).

However, one must consider the implications of repeated use of MNs, particularly dissolving MNs, where deposition of polymer in the skin from the dissolving system is undesirable. For example, the dissolving MN used in the McCrudden et al. study (126) would deposit approximately 5–10 mg of polymer per cm^2^ in the skin. Assuming a patch size of 10 cm^2^, 50–100 mg of polymer would be deposited into the skin each time a patch is applied. This is unlikely to be a concern for vaccinations, however, most therapeutic agents, particularly biologics discussed in this review, require repeated administration. This supports the ongoing use of hydrogel-forming MNs (Fig. 1E) as the MN device can be removed from the skin intact, without polymer deposition. Dissolving MNs may be better placed for vaccine delivery, as their infrequent use reduces the issue of repeated polymer deposition within the skin.

In vivo studies revealed that repeat application of both dissolving (once daily for 5 weeks) and hydrogel-forming (twice daily for 3 weeks) MNs did not alter skin appearance or barrier function and caused no measurable disturbance of serum biomarkers of infection, inflammation or immunity (132)*.* More recently, the clinical impact of repeated application of hydrogel-forming MN arrays was assessed by Al-Kasasbeh et al. (2020).The authors of this study repeatedly applied a hydrogel-forming MN array to the upper arm of human volunteers over a 5 day period. Safety of repeated MN application was assessed by measuring skin barrier integrity and the presence of systemic inflammatory biomarkers (C-reactive protein, interleukin 1-β, tumour necrosis factor-α, immunoglobulin G and immunoglobulin E) in blood. The results demonstrated that repeat hydrogel-forming MN application does not lead to prolonged skin reactions or prolonged disruption of skin barrier function.

Furthermore, concentrations of systemic inflammation biomarkers were all found to be within the normal range (133). It appears as though MNs may cause adverse events (such as skin irritation and intradermal granulomas) only when used inappropriately, i.e., when used in combination with cosmetic products that were not intended for application to MN-punctured skin (134,135).

However the question still remains, could repeated delivery of protein, peptide and antibody based therapies eventually result in an immune response from the host? Proteins and peptides present in infusions may trigger an immune response if the body recognises them as antigens (12,13), and the same may be the case for MN mediated delivery. Although biological therapies are typically designed to be non-immunogenic via “humanisation”, delivery via the dermal route allows the drug to come into contact with a wealth of immune cells involved in the skin’s innate immune response, namely Langerhans cells in the epidermis and dendritic cells in the dermis (136,137). These immune cells are present in much higher concentrations than those in subcutaneous tissue or muscle, the traditional target of protein based drugs. One study focused on the delivery of ovalbumin-loaded PLGA nanoparticles via electrohydrodynamic coating of MNs (138). In addition to showing extended release of ovalbumin over 28 days, the study also explored possible immunogenic effects of delivery of ovalbumin into dermal tissue. The coated MNs resulted in no significant increase in anti-OVA-specific IgG titres in C57BL/6 mice in vivo as compared to the untreated mice (*p* > 0.05), indicating that the formulations are nonimmunogenic.

Whilst it seems unwanted immunogenic effects from protein and peptide drugs delivered via MNs are unlikely, long-term studies exploring the immune response following repeated application of MNs containing biological drugs are required before clinical acceptance can be assured, and will likely have to be shown on a drug-by-drug basis.

### Patient/Prescriber Acceptability

The success of MN based products is also dependent on the acceptability to patients and healthcare professionals. A prescriber must be willing to prescribe the product, and patients must accept the product and be able to apply the MN array correctly. Patient benefits, including reduced pain, blood, and needle stick injuries, increased acceptability by people with needle phobia and the potential for self-administration were the most important factors influencing the opinion of MNs in various groups such as in children and the elderly (66,139,140). Mild erythema post MN removal may be a concern for some patients, however barrier function will recover within hours and skin reddening will be transient (56). It is imperative for prescribers to properly educate users of MN devices, to prevent effects such as mild erythema from reducing patient compliance.

Appropriate MN application is of particular importance for cases such as global pandemics or bioterrorism incidents, where necessary treatment may be dependent on the ability to self-apply the device. Previous studies have shown that patients can successfully apply MNs to their own skin following instruction provided by pharmacist counselling in conjunction with a patient information leaflet (68). Furthermore, a “dosing indicator” has been developed to assure patients that application was successful (141). This is alongside appropriate instructions (68,142). This may be of particular use in the elderly, where declining motor function and manual dexterity may be an issue (143).

Qualitative studies demonstrate the complex and multifaceted nature of end-user acceptance. Thus, such studies will undoubtedly aid industry in taking the necessary action to address concerns and develop informative labelling and patient counselling strategies to ensure safe and effective use of MN devices. Marketing strategies will also be vital in achieving maximum market share relative to existing and widely accepted conventional delivery systems.

### Regulatory Authority Acceptability

The likely considerations and potential requirements from a regulatory standpoint that must be addressed for MNs to be accepted for clinical use are summarised in Table II. One of the greatest concerns moving forward is whether MNs will be accepted as a drug delivery system, consumer product, or medical device. If MNs are to be considered closer to a traditional hypodermic injection than a transdermal patch, regulatory authorities are likely to request that the device is rendered sterile prior to use. Aseptic manufacture will be expensive and will present practical challenges if large-scale manufacturing is required. Furthermore, gamma irradiation, moist heat or microwave heating may damage the MNs or biomolecule cargoes, contaminating the delivery system. For example, McCrudden et al. (126) found that gamma radiation significantly reduced drug content (ovalbumin and ibuprofen) in dissolving MNs and the lyophilised wafer-type drug reservoirs, although the hydrogel-forming MNs (without drug) were unaffected (144). As equivalent manufacturing techniques are not currently available, any manufacturer wishing to develop MN products will need to make a substantial initial capital investment. Specifically for protein, peptide and antibody based therapies, stability of the formulation and potential immunological effects will be of particular concern.

**Table II:** The likely considerations and potential requirements from a regulatory body that must be addressed for MNs to be accepted for clinical use. Replicated with permission from (131)

Sterility of the MN dosage form	MNs penetrate the skin surface rather than adhering to it as would a traditional transdermal patch MNs may be required to be rendered sterile depending on regulatory considerations A low bioburden may be sufficient if the system has inherent and demonstrable antimicrobial activity
Uniformity of content	Either from the system as a whole, or potentially of individual drug loaded MNs within an array, depending on the system design Likely required as is the case with all other conventional transdermal patch dosage forms
Packaging	Security of packaging, i.e., protection from water ingress Ease of removal from packaging by patients without accidental piercing of the skin prior to intended application
Potential for MN re-use	Certain MN devices may be removed intact from the skin with the potential to re-pierce the skin e.g. silicon MNs Dissolving or hydrogel-forming MNs will likely be preferred as they are self-disabling
Disposal procedures	MN materials that are not dissolvable or biodegradable may be a hazard Environmental aspects of disposal must be considered
Deposition of MN material into skin	Of particular concern with dissolving MNs and those devices which would be used for chronic conditions Product may require alternating application site Potential for short term adverse effects, such as granuloma formation or local erythema, must be stated
Ease and reliability of MN application	Patients must be able to use the product properly, without significant inconvenience
Assurance of MN insertion	Indication of correct application and delivery (particularly for vaccination applications) may be required Would be useful to assure patients they have applied the device correctly
Potential immunological effects	Repeated insult of the skin, an immunologically active site, by MNs may result in an immunological reaction Assurances regarding immunological safety will be required

These issues are somewhat intensified when considering the need for scale up manufacture of MNs, as the large scale requires further investment to overcome issues associate with formulating biologics alongside, and within, MNs. Laboratory-based processes are often difficult to scale-up initially, with problems of cost-efficiency of mass manufacture and turnaround time (145). Turnaround time will be dictated by Good Manufacturing Practice (GMP), Quality Assurance (QA) and Quality Control (QC) guidelines – the more stringent, the longer the turnaround time. These guidelines will also be influenced by the classification of MNs, discussed above. Rapid turnaround of MNs containing biologics, particularly vaccines, may be required during a pandemic, like severe acute respiratory syndrome (SARS) in 2003, the influenza H1N1 outbreak in 2009, and the coronavirus Covid-19 pandemic in 2020.

QC tests differ from those that are presented in Table II as they refer to those tests that might be performed during the manufacture of either the drug substance or drug product (146). Furthermore, QC additionally refers to the organisation, documentation and release procedures designed to ensure that the necessary and relevant tests are carried out, and that materials are not released for use, nor products released for sale or supply, until their quality has been judged satisfactory (145). However, without an understanding of the classification and acceptance criteria of MN arrays (speculated in Table II), one can only speculate on the QC tests required.

One must ask the question therefore: what are the basic requirements of MNs? The answer to this question dictates the acceptance criteria, and thus the QC tests which must be undertaken to assure the manufacturers that the MNs are meeting this basic requirement. MNs must adequately pierce the skin, penetrate, and be able to be removed intact. Where dissolving MNs are used, they must be able to sufficiently release their drug cargo within a reasonable period. Hollow MNs must remain “open” for the duration of drug delivery, and hydrogel-forming MNs must swell appropriately for delivery of the drug through the associated drug cargo. The MNs must not harm the patient. Lutton et al. (145) demonstrated that MNs fall within the scope of the International Conference of Harmonisation (ICH) Q6A guidelines (146) and used these criteria to set out a list of quality specifications applicable for all MNs. Therefore, QC tests that are likely to be performed on MNs during the manufacturing process include dissolution, disintegration, friability, uniformity of dosage, stability, water content where appropriate, microbial limits, sterility (if required by the manufacturer), particulate matter, antimicrobial preservative content, extractables, functionality of delivery system, and osmolarity. Furthermore, mechanical testing of MNs will be required – they must be hard enough to pierce the skin, but without being brittle, so as not to fracture, leaving the needle within the skin. Such tests include force of MN insertion/bending/fracture (i.e. axial, transvers, fracture force); insertion tests to ensure patients can manually insert the needles successfully, given that patients may apply needles over a range of forces and they cannot “calibrate” their application force; confirmation of MN insertion (i.e. via OCT (147)); and tests to ensure skin barrier function returns to baseline rapidly following MN removal (i.e. TEWL/TEER). Not only are such tests required, but a reasonable range of expected analytical and manufacturing variability must also be considered where appropriate. Even with these tests in place, further questions remain, such as, what is an appropriate model membrane to test for sufficient MN insertion? Skin cannot be used for QC testing, therefore any model membrane must be able to replicate the skin structure, which is challenging given that skin is not homogeneous.

Despite the ongoing questions surrounding MN manufacture and acceptance, it is interesting to note that the US FDA recently published draft guidance on “microneedling” for cosmetic applications (148), and PATH recently released a fact sheet illustrating a four-year initiative for accelerating the development of MNs for drug delivery and vaccines (149). Thus, the interest of regulators in the technology is clearly presented.

## Conclusion

To conclude, numerous studies have illustrated the ability to deliver therapeutic doses of protein, peptide and antibody based therapies using MNs as an alternative drug delivery device. The advantages of MNs over traditional routes of drug delivery are apparent, and thus, it appears likely that MNs will be used as a drug delivery device within the next ten years. The use of these devices could vastly improve the quality of life for patients, improve public health, and increase the economic productivity of developing countries.

Future success of MNs will be contingent on their long-term safety profile, methods of manufacture, and their ability to comply with standardised GMP guidelines. Furthermore, marketing strategies will also be vital in achieving maximum market share relative to existing and widely accepted conventional delivery systems. In the meantime, academia and industry must work together to address concerns, and thereby push MN technology into the clinic, where its potential can be truly realised.

### Expert Opinion

At present, MNs are primarily viewed as vaccine delivery systems for the developing world despite a plethora of published articles demonstrating their ability to deliver of a wide range of therapeutic molecules. Consequently, many pharmaceutical companies are unwilling to make investments in the field. For this reason, it is vitally important to change this mind set by proving that MNs offer far more than acting as simple vaccine delivery devices. Indeed, MNs have made important advancements in removing the need for needle and syringe in the treatment of diseases such as HIV, diabetes, Alzheimer’s and cancer (85,88,111,150,151). However, translating these findings from bench-top to bedside is proving extremely challenging. More recently, MNs have been tested for diagnostic fluid sampling (152,153). Evidently, this field has huge potential, with the possibility of MN sampling devices being delivered to patients’ homes and returned to the laboratory for analysis, without the patient having to enter a clinical setting. Perhaps, with further optimisation, a MN sampling device could be used in viral testing and, as a result, prove instrumental in the fight against future pandemics. As this MN design could be CE marked as a medical device rather than a drug product, reduced regulatory requirements could mean that pharmaceutical companies may be willing to first invest in a device such as this. Importantly, the initial upfront costs of scaled up manufacture could be offset by achieving faster market commercialisation. As a result, the infrastructure would be in place for the manufacture of drug containing MNs in the future.

Moving forward, pharmaceutical companies must view drug containing MNs as commercially viable. One way to achieve this is through focussed collaboration between academia, industry, healthcare professionals and patients. In particular, it is vitally important that the views and opinions of patients are respected and considered fully, as many products have failed in the past as they have forgotten about the end user. In this regard, MNs have shown to help overcome needle phobia through painless application, thus improving patient compliance (65,66). As a result, MN-mediated administration has the ability to reduce the frequency of repeat hospitalisation through minimising the number of missed doses. Furthermore, dissolving and hydrogel-forming MNs are designed to prevent needle re-use, to reduce the likelihood of needle-stick injury and to remove the need for dedicated sharps disposal. Therefore, the substantial benefits to both the patient and health care sector could outweigh the initial scale-up costs incurred by the industry. To this end, Zosano Pharma have developed a zolmitriptan based intracutaneous MN system for the treatment of migraine headaches. This MN device successfully produced a therapeutic effect in clinical studies and as result, is expected to receive FDA approval in 2021. As the first drug containing MN product to potentially achieve commercialisation, it appears many pharmaceutical companies are willing to delay MN development until its success becomes apparent. However, it is anticipated that this novel device will offer additional benefits to both patients and healthcare providers, thus opening the door for the next generation of transdermal delivery systems.

#### ACKNOWLEDGMENTS AND DISCLOSURES

Aaron R.J. Hutton is a PhD candidate funded by Department for the Economy (N. Ireland) studentship. Additionally, the authors would like to express their sincere gratitude to William Kerr for his assistance with the creation of Fig. I.

## Abbreviations

MN: Microneedle
